# Outcomes of Hematopoietic Stem Cell Transplantation in 5 Patients with Autosomal Recessive RIPK1-Deficiency

**DOI:** 10.1007/s10875-024-01850-2

**Published:** 2025-01-06

**Authors:** Rebecca B. Walsh, Peter McNaughton, Zohreh Nademi, Alexandra Laberko, Dmitry Balashov, Hamoud Al-Mousa, Peter D. Arkwright, Robert F. Wynn, Terry Flood, Eleri Williams, Andrew Cant, Mario Abinun, Sophie Hambleton, Mary Slatter, Andrew R. Gennery, Su Han Lum, Stephen Owens

**Affiliations:** 1 Royal Victoria Infirmary, Newcastle-Upon-Tyne, UK; 2 Population Health Sciences Institute, Newcastle University, Newcastle-Upon-Tyne, UK; 3 Great North Children’s Hospital, Newcastle-Upon-Tyne, UK; 4 Translational and Clinical Research Institute, Newcastle University, Newcastle-Upon-Tyne, UK; 5 Hematopoietic Stem Cell Transplantation, Dmitry Rogachev National Medical Research Centre of Paediatric Haematology, Oncology and Immunology, Moscow, Russia; 6 Section of Paediatric Allergy and Immunology, Department of Paediatrics, King Faisal Specialist Hospital and Research Centre, Riyadh, Saudi Arabia; 7 Lydia Becker Institute of Immunology and Inflammation, University of Manchester & Royal Manchester Children’s Hospital, Manchester, UK; 8 Blood and Marrow Transplant Unit, Royal Manchester Children’s Hospital, Central Manchester University Hospitals NHS Foundation Trust, Manchester, UK

**Keywords:** Autosomal recessive receptor interacting serine/threonine kinase 1 deficiency, hematopoietic stem cell transplant, primary immunodeficiency, inborn error of immunity

## Abstract

Receptor Interacting Serine/Threonine Kinase 1 (RIPK1) is widely expressed and integral to inflammatory and cell death responses. Autosomal recessive RIPK1-deficiency, due to biallelic loss of function mutations in *RIPK1*, is a rare inborn error of immunity (IEI) resulting in uncontrolled necroptosis, apoptosis and inflammation. Although hematopoietic stem cell transplantation (HSCT) has been suggested as a potential curative therapy, the extent to which disease may be driven by extra-hematopoietic effects of RIPK1-deficiency, which are non-amenable to HSCT, is not clear. We present a multi-centre, international review of an additional 5 RIPK1-deficient children who underwent HSCT. All patients presented with very early onset inflammatory bowel disease, 2 also suffered from inflammatory arthritis. Median age at transplant was 3 years (range 1—5 years); 1 received matched sibling marrow, 1 matched unrelated peripheral blood stem cells (PBSC), 2 TCRαβ/CD19-depleted PBSC from maternal-haploidentical donors, and 1 had TCRαβ/CD19-depleted PBSC from a mismatched unrelated donor. All received reduced-toxicity conditioning, based on treosulfan (*n* = 4) or busulfan (*n* = 1); 1 patient underwent a successful second transplant following autologous reconstitution. Four of five patients (80%) survived; 1 child died due to multi-drug resistant pseudomonas infection and multi-organ failure. With a median duration of 14 months follow-up, 2 survivors were disease-free, and 2 had substantially improving enteropathy. These findings demonstrated that HSCT is a potential curative therapy for RIPK1-deficiency.

## Introduction

Receptor-interacting serine/threonine-protein kinase 1 (RIPK1) is a key molecular mediator of apoptotic and necroptotic cell death and of inflammatory signaling-pathways. Impairments of the scaffolding and kinase-specific functions of RIPK1, implicated in the pathogenesis of conditions which are characterised by severe immune dysfunction associated with cytokine dysregulation, have recently been reviewed [1, 2].

Biallelic loss of function (LOF) mutations in *RIPK1* cause severe immunodeficiency in humans, characterised by impaired T- and B-lymphocyte differentiation, lymphocytopenia and diminished production of interleukin (IL)−6, tumour necrosis factor (TNF) and IL-12. Despite the deficit in these pro-inflammatory cytokines, there is a severe inflammatory component to RIPK1-deficiency immunodeficiency with autoinflammation [3], thought to be a function of enhanced activation of inflammasomes and over-production of IL-1β [1, 3–6]. The resulting phenotypes of RIPK1-deficiency have been summarised for 16 patients from among 13 families to date, and comprise inflammatory bowel disease (IBD), recurrent viral, bacterial and fungal infections, and, in some cases, progressive, inflammatory polyarthritis [3, 7–10]. In contrast, dominantly-inherited, heterozygous missense mutations in *RIPK1* generate a protein which resists caspase cleavage, promoting its ongoing activation and leading to recurrent fevers and lymphadenopathy – a condition known as cleavage-resistant RIPK1-Induced autoinflammatory disease (CRIA) [2].

While IL-1β blockade merits further evaluation in patients affected by LOF mutations leading to RIPK1 deficiency [3], only hematopoietic stem cell transplant (HSCT) offers a potential cure [1]. The outcomes of 4 children who underwent HSCT for RIPK1-deficiency, 3 of whom died, have been previously reported [3, 7]. To the best of our knowledge no patients with CRIA have yet been transplanted. To better understand the potential role of HSCT as a curative treatment for RIPK1-deficiency, we report on the outcome of 5 additional patients with biallelic LOF mutations in *RIPK1*, transplanted between 2019 and 2021.

## Patients and Methods

Patients were identified from the Stem Cell Transplant for Primary Immune Deficiencies in Europe (SCETIDE) Registry for patients. A standardized proforma was sent to respective transplant centres to retrieve relavant data for this study. Using the proforma the following data were extracted: patient demographics, *RIPK1* mutation, disease characteristics, treatments prior to HSCT, transplantation characteristics, transplant outcomes, long-term graft function and disease outcomes. Informed consent was obtained from legal guardians as per institutional practice.

## Results

### Patient Characteristics

We identified 5 children (4 females) from 4 families transplanted in 3 centres between 2019 and 2021; 3 at The Great North Children’s Hospital, United Kingdom (UK) (N1-N3), 1 at King Faisal Specialist Hospital & Research Centre, Saudi Arabia (N4), and 1 at Dmitry Rogachev National Medical Research Centre of Paediatric Haematology, Oncology and Immunology, Russia (N5). Patient characteristics are shown in Table 1. The pre-HSCT course of N1 was featured in the earlier case series by Cuchet-Lourenço et al. [3]. Several genetic variants were identified as causing the biallelic LOF of *RIPK1,* all of which were previously reported as being pathogenic [3, 8]. All patients presented with very-early onset IBD (VEOIBD); median age of onset was 1.5 months (0.7 – 5.0 months); N1-N3 had recurrent infections and went on to develop inflammatory arthrits by 2 years of age; N5 had one episode of bacterial lymphadenitis prior to transplant.

**Table 1:** Clinical characteristics

Patient	Family	Centre	Year of birth	Sex	RIPK1 mutation	Disease Characteristics					
						IBD (Age on onset months)	Arthritis (Age on onset years)	Lymphopenia (T, B, NK cells)	Infections	Failure to thrive (Intervention needed)	Other
N1	A	GNCH	2013	F	Deletion of 2064bp in RIPK1 c.460–133_688 + 1702del [3]	4	Small joints of hand + Knees 2	T, B cells	Bronchiolitis; *P. aeruginosa* sepsis; Perianal and gut candida; Otitis media	Y- TPN + PEG	Perianal abscess; Perianal ulceration; Anal fistulas
N2	A	GNCH	2016	F	Deletion of 2064bp in RIPK1 c.460–133_688 + 1702del [3]	1	Polyarthritis 1.7	T, B cells	Bronchiolitis; *Pseudomonas aeruginosa* and *Staphylococcus epidermidis* sepsis	Y-TPN	Hepatosplenomegaly; Bone marrow myelofibrosis; Peri-anal ulceration; Gross motor developmental delay; Cholecystectomy
N3	B	GNCH	2016	F	Microdeletion: c.688_688 + 20del [3]	5	Small joints of hand + Knees 2	T, NK cells	Recurrent oral candida; Disseminated salmonella	Y- TPN	Genital abscesses; Recto-vaginal fistula; Delayed motor skills
N4	C	KFSH&RC	2018	M	Missense c.1934C˃T: p. Thr645Met [8]	0.7	N	N	N	Y- Unknown	
N5	D	DRNMRCPHOI	2019	F	Missense c.1934C > T, p. Thr645Met [8]	1.5	N	N	Bacterial lymphadenitis	Y – PPN	Severe fistulating enteropathy; ileostomy; Perianal abscess; Perianal ulceration; Mild dermatological involvement

*CMV,* Cytomegalovirus; *DRNMRCPHOI,* Dmitry Rogachev National Medical Research Centre of Paediatric Haematology, Oncology and Immunology; *F,* Female; *GNCH,* Great North Children’s Hospital; *HSV,* Herpes simplex; *KFSH&RC,* King Faisal Specialist Hospital and Research centre; *IBD,* inflammatory bowel disease; *M,* Male; *MRSA,* Methicillin-resistant staphylococcus aureus; *N,* No; *NG,* Nasogastric; *NK,* Natural killer; *P,* Patient; *PEG,* Percutaneous Endoscopic Gastrostomy; *RIPK1,* Receptor Interacting Serine/Threonine Kinase 1; *RSV,* Respiratory syncytial Virus; *TPN,* Total parenteral nutrition; *PPN,* Partial parenteral nutrition; *VZV,* Varicella zoster virus

Prior to HSCT, all patients were immunosuppressed with combinations of sulfasalazine, azathioprine, infliximab, rituximab and steroids for their IBD, with minimal responses. N1 had several doses of anakinra, however repeat endoscopies revealed worsening ulceration following its introduction, with widespread candida detected in the upper and lower gastrointestinal tracts, hence the treatment was withdrawn. N2 (younger sister of N1) presented with inflammatory enteropathy, which was particularly severe and proved impossible to control with steroids and infliximab. In the light of the experience with her sister however, treatment with anakinra was not attempted. N5 responded well to anakinra for her colitis, although she continued to have inflammatory changes in the recto-sigmoid so treatment was continued throughout the peri-transplant period. All patients presented with growth failure and 4 required parenteral nutrition pre-HSCT. All except N4 received intravenous immunoglobulin replacement prior to transplant. N5 had direct antiglobulin-positive haemolytic anaemia pre-HSCT, which responded to rituximab.

### Transplant Characteristics and Outcome

A detailed description of transplant characteristics is summarised in Table 2. The median age at transplant was 2.6 years (range 1.2 – 5.0 years). Median interval between presentation and transplant was 2.5 years (range 1.1 – 4.8 years). Donor and stem cell sources were matched sibling donor (MSD) marrow (*n* = 1), matched unrelated donor (MUD) peripheral blood stem cells (PBSC) (*n* = 1), TCRαβ/CD19–depleted maternal haploidentical donor PBSC (*n* = 2) and mismatched unrelated donor PBSC (*n* = 1).

**Table 2:** Transplant details

Patient/Centre/Year		Age at HCT (years)	Time from presentation to HCT (years)	Donor Sex and Age (years)	Donor	Stem cell source	Conditioning	Graft CD34 × 10^6^ /kg	GvHD prophylaxis	Engraftment	Latest donor Chimerism	Outcome (FU- in Years)
N1- GNCH 2019		5.0	4.67	Male—21	MUD	PBSC	Fludarabine 150 mg/m^2^ Treosulfan 42 g/m^2^ Alemtuzumab- 1 mg/kg	38.2	CSA/MMF	Neutrophil D + 12 Platelet D + 14	CD15 + 100% CD3 + 96%	Alive and well (1.8)
N2- GNCH 2019		2.6	2.52	Female—29	Maternal HID TCRαβ/CD19 depleted	PBSC	Fludarabine 160 mg/m^2^ Thiotepa 10 mg/kg Treosulfan 42 g/m^2^ Anti-thymocyte globulin 15 mg/kg Rituximab 200 mg/m^2^	15.5	None	Neutrophil D + 10 Platelet D + 16	CD15 + 100% CD3 + 100%	Fed via percutaneous endoscopic gastrostomy, on immunoglobulin weekly (0.4)
N3- GNCH 2021		5.0	4.58	Female—33	Maternal HID TCRαβ/CD19 depleted	PBSC	Fludarabine 160 mg/m^2^ Thiotepa 10 mg/kg Treosulfan 36 g/m^2^ Anti-thymocyte globulin 15 mg/kg Rituximab 200 mg/m^2^	7.2	None	Neutrophil D + 14 Platelet D + 18	WB:100%	Death Day + 61 Sepsis Multi-organ failure
N4- KFSH&RC 2020		1.2	1.1	Male – 6	MSD	Bone marrow	Busulfan AUC 60–80 mg/Lhr Fludarabine 150 mg/m^2^ Anti-thymocyte globulin 15 mg/kg	6.7	CSA /Methotrexate	Neutrophil D + 13 Platelet D + 31	CD15: 100% CD3: 100%	Alive and well (1.6)
N5- DRMRCPHOI 2020	1st transplant	1.2	1.14	Male – 52	MMUD TCRαβ/CD19 depleted	PBSC	Fludarabine 150 mg/m^2^ Treosulfan 42 g/m^2^ Cyclophosphamide 100 mg/kg Anti-thymocyte globulin 15 mg/kg Rituximab 200 mg/m^2^	17.1	None	Neutrophil D + 10 Platelet D + 10	Graft rejection at 6 months post-HCT	Second transplant
	2nd transplant	2.2		Male – 53	MMUD TCRαβ/CD19 depleted	PBSC	Fludarabine 150 mg/m^2^ Treosulfan 42 g/m^2^ Melphalan 140 mg/m^2^ Anti-thymocyte globulin 15 mg/kg Rituximab 200 mg/m^2^	14.16	None	Neutrophil D + 17 Platelet D + 10	WB: 100%	Alive; off immunoglobulin replacement; remains on prophylactic antibiotics and requires dietary modulation and mesalazine therapy for colitis + perianal dermatitis (1.9)

*CD,* cluster of differentiation; *CSA,* cyclosporine A; *DRNMRCPHOI,* Dmitry Rogachev National Medical Research Centre of Paediatric Haematology, Oncology and Immunology; *GvHD,* graft versus host disease; *HID,* haploidentical donor; *GNCH,* Great North Children’s Hospital; *KFSH&RC,* King Faisal Specialist Hospital and Research Centre; *MMF,* mycophenolate mofetil; *MUD,* matched unrelated donor; *MMUD,* mismatched unrelated donor; *MSD,* matched sibling donor; *PBSC,* peripheral blood stem cells; *TCR,* T cell receptor; *WB,* whole blood

All received reduced toxicity conditioning regimen, which varied accordingly to transplant. The 3 patients who were transplanted in the UK (N1-3) received fludarabine-treosulfan, with additional thiotepa, anti-thymocyte globulin (ATG) and rituximab in the TCRαβ/CD19–depleted haploidentical donor transplants [11], and alemtuzumab in the MUD transplant. N4 was conditioned with busulfan, fludarabine and ATG for MSD marrow transplant. N5 had fludarabine-treosulfan-cyclophosphamide with ATG and rituximab. The median times to neutrophil and platelet engraftment were 12 days (range 10–17) and 15 days (range 10–31) respectively. N1-N4 achieved full donor chimerism (> 95% in all cell lines). Median duration of follow-up of surviving patients was 1.5 years (range 0.4–1.9 years).

N1’s peri-transplant course was complicated by an *E. coli* bacteraemia and disseminated adenovirus infection (positive adenoviral PCR in blood, faeces and naspharyngeal aspirate), successfully treated with cidofovir and subsequently with brincidofovir. Complete disease resolution was achieved by 4 months post-transplant. At last available follow-up (1.8 years post transplant) she had full donor chimerism, was off all medication and was thriving on a normal diet. She has subsequently been lost to follow-up.

N2 (younger sister of N1) developed veno-occlusive disease on Day + 6 which resolved with defibrotide. Her recovery was complicated by perianal fistulating enteropathy, secondary to her severe colitis pre-transplant. Despite apparently robust immune reconstitution, she had a late re-admission with sepsis, the focus of which was presumed to be gut translocation. At the most recent follow-up (5 months post-HSCT), she remained on intravenous immunoglobulin. N2 had some residual but quiescent perianal disease and, despite optimal enteral feeding support, remained below the 0.4th percentile weight-for-age. Like her sister, she has subsequently been lost to follow-up.

N3 developed a vesicular rash from Day + 39 which eluded histological diagnosis. No viruses were identified and skin biopsy was suggestive of grade IV graft versus host disease (GVHD) or toxic epidermal necrosis. She died of multiorgan failure secondary to a multi-drug resistant pseudomonas septicaemia on Day + 61 post-HSCT.

N4 was treated for cytomegalovirus and adenovirus viremias and his post-HSCT course was complicated by sepsis and pulmonary hemorrhage. However, at the most recent follow-up appointment, he had complete symptom resolution.

N5 developed secondary autologous reconstition at 6 months and required a second transplant from the same donor, with melphalan replacing cyclophosphamide in the conditioning regimen. Full donor chimerism was achieved by Day + 30 after second transplant. At 1.7 years, she developed rash which was interpreted as late-onset aGVHD. This was sucesfully treated with ruxolitinib. At 1.9 years post-transplant, she had good lymphoid reconsitution with full donor chimerism and was off immunoglobulin replacement. She remained on mesalazine with low weight-for-height and mild fecal incontinence, but otherwise had no symptoms of active gut disease.

## Discussion

We report transplant outcomes of 5 additional children with autosomal recessive RIPK1-deficiency, who underwent HSCT in 3 centers in UK, Saudi Arabia and Russia between 2019 and 2021. Similarly to previous reports of RIPK1 deficiency, the patients all presented with diarrhea and growth failure secondary to IBD and 3 of them also developed recurrent infections and arthritis by the time they were referred to a transplant center. Attempts at disease-modification pre-HSCT with steroids, TNF-blockade and other biologics were largely ineffective in our cohort.

There was no established treatment pathway for affected patients prior to transplant. We attempted IL-1 blockade with anakinra in 2 of our patients following descriptions of exaggerated IL-1 production by affected monocytes in previous reports [3, 7]. Treatment at a dose of 6 mg/kg preceded deterioration in the colitis of N1, with ulceration and super-added candida infection, and was stopped after 6 weeks. There was no appreciable effect on joint inflammation. In contrast, anakinra was thought to have reduced the severity of gut disease in N5 at a dose of 7 mg/kg. A previous case study reported the use of anakinra (3 mg/kg) in a patient with RIPK1-deficiency and refractory colitis but the response was not documented [10]. Much higher doses of anakinra up to 10 mg/kg were effective in suppressing intestinal inflammation in 2 patients with deleterious *IL10A* mutations, which raises the possibility of a dose-dependent effect which we did not achieve with the regimen we employed [12].

Patients from our series were transplanted between 1 and 5 years after initial presentation. Patient N3 died aged 5 years from overwhelming infection, 2 months after HSCT. The remaining 4 exhibited complete donor chimerism at last follow-up (0.5 – 1.9 years post-HSCT). Successful HSCT led to complete resolution of symptoms in 2 patients, and improvement in gut symptoms in the other 2. N2 had particularly severe fistulating IBD pre-transplant and continued to suffer with this problem at last follow-up, 4-months later. Other than confirming that she remains alive, it has not been possible to obtain further information about her condition at the time of writing, as she returned to her home country and has been lost to follow-up. A prolonged recovery period might be expected after such severe pre-existing tissue damage. Her sister (N1), by contrast, had complete resolution of disease-related symptoms at 1.8 years post-transplant. N5 still has some mild gut problems with suboptimal growth and takes mesalazine. She has good lymphoid reconstitution and has recently come off immunoglobulin replacement. She remains on prophylactic antibiotics, 1.9 years post-HSCT.

Cuchet-Lourenço et al. reported 3 children who had undergone HSCT by the time of publication in 2018 2 of whom were also transplanted at GNCH [3]. While 1 underwent a successful transplant and was living a disease-free life, the other 2 children died from transplant-related complications. One developed capillary leak at Day −2 and multi-organ failure with encephalopathy from Day + 7; he died on Day + 46. The other child reactivated adenovirus and *Herpes simplex* on Day + 14 and Day + 29 respectively and died on Day + 33 from multi-organ failure and catastrophic pulmonary haemorrhage. In the other published report of a patient transplanted with RIPK1-deficiency, few details were given: the molecular diagnosis was made post-mortem; the patient was said to have been symptomatic with recurrent infections from birth and received a matched-family donor HSCT at 12 months of age; the graft failed and she was re-transplanted using the same donor at 3 years 2 months; she eventually died aged 19 years from chronic GVHD and ‘pulmonary disease’ [7].

Therefore, of 9 patients with RIPK1-deficiency known to have been transplanted to date, 4 have died (44%). Such high overall transplant-related mortality may in part reflect the uneven age-distribution of this small cohort, skewed by those older than 5 years in earlier reports, who are generally at higher risk of death post-HSCT. Other transplant series for IEI characterized by VEOIBD showed that older age was associated with inferior transplant survival, likely reflecting more established pre-transplant organ damage and recrudescent viral infections [13, 14]. Moreover, the pro-inflammatory nature of the condition, apparently resistant to steroids and biologic immuno-modulation pre-HSCT, is likely to increase the risks.

By replacing RIPK1-deficient host hematopoietic stem cells with healthy donor cells, it has already been shown that cytokine production and immune function can be normalized in HSCT survivors [3]. However, it was unclear whether HSCT could resolve all the manifestations of RIPK1-deficiency in all tissues or whether symptoms might relapse with non-canonical necroptosis in non-hematopoietic cells, or by dysregulated inflammation in long-lived tissue-resident lymphoid cells, which had escaped pre-HSCT conditioning [1]. This possibility was illustrated by recent transplant experience with Nuclear Factor κB Essential Modulator (NEMO) defects, in which HSCT appeared to correct the function of immune cells but not of intestinal epithelial cells, accounting for the persistence of colitis after transplant in some patients and the new onset of colitis post-HSCT in others [12]. However, the evidence presented here suggests that such concerns may be misplaced in patients transplanted for RIPK1 deficiency. Moreover, this case series again demonstrates how graft manipulation in mismatched family or unrelated donors allows affected children to be successfully transplanted, when they otherwise lack a suitably matched donor [15]. Reduced toxicity conditioning using either treosulfan or busulfan was well tolerated despite multi-system organ damage prior to HSCT.

There are insufficient data with which to properly compare outcomes between those undergoing HSCT for RIPK1 deficiency and those treated conservatively. Of 11 patients who had not been transplanted at the point that their cases were published, 3 are known to have died (27%) [7, 8] while outcomes for another 2 were not clearly reported [7, 9]. Of the rest, little clinical information was given about their clinical status beyond descriptions of unsuccessful treatment with various immuno-modulators for refractory IBD [7, 8, 10]. The ages of surviving patients at last clinical follow-up are likewise unclear from these reports, however they were aged between 16 months and 10.5 years at molecular diagnosis and since they were diagnosed with a novel IEI, this was likely to have been shortly before publication [7, 8, 10].

## Conclusion

In conclusion, autosomal recessive RIPK1-deficiency is characterized by severe VEOIBD, growth failure and recurrent infections with lymphopenia in most patients, while other features such as inflammatory arthritis are more variable. The disease appears to be unresponsive to various forms of immunomodulation and carries a substantial risk of sepsis and death in childhood. On the other hand, the evidence provided by previous reports and now supported by this larger case series, suggests that successful HSCT is curative in 55.5% of patients (5/9) overall, and 80% (4/5) in our more recent series. Moreover, life expectancy and especially quality of life in children who survive transplant is likely to be much better than those who are treated conservatively [16], and this prognosis is also supported by the findings from the current study at latest follow-up. On this basis, we propose that serious consideration be given to offering HSCT to children with RIPK1 deficiency, soon after diagnosis, ideally at a specialist center with experience transplanting the condition. However, we acknowledge that the overall risk of transplant-related mortality appears to be higher than for ‘classical’ primary immunodeficiency disorders (PID), as is also reported for other IEI with complex underlying pathophysiology, such as NEMO and the various Hyper IgE syndromes, making the decision to offer HSCT at diagnosis particularly difficult [17]. Therefore, the potential role of targeted anti-inflammatory treatment such as IL-1 blockade in the pre-or peri-transplant period requires further study.

## Data Availability

No datasets were generated or analysed during the current study.
